# Clozapine in Treatment-Resistant Schizophrenia and Its Augmentation with Electroconvulsive Therapy in Ultra-Treatment-Resistant Schizophrenia

**DOI:** 10.3390/biomedicines11041072

**Published:** 2023-04-02

**Authors:** Vjekoslav Peitl, Antonia Puljić, Mislav Škrobo, Sergej Nadalin, Lidija Fumić Dunkić, Dalibor Karlović

**Affiliations:** 1 Department of Psychiatry, University Hospital Centre Sestre Milosrdnice, 10000 Zagreb, Croatia; 2 School of Medicine, Catholic University of Croatia, 10000 Zagreb, Croatia; 3 Department of Psychiatry, General Hospital “Dr. Josip Benčević”, 35000 Slavonski Brod, Croatia; 4 Department of Anesthesiology, Intensive Care and Pain Therapy, University Hospital Centre Sestre Milosrdnice, 10000 Zagreb, Croatia

**Keywords:** clozapine, ECT, TRS, UTRS

## Abstract

Clozapine is considered the gold standard for patients with treatment-resistant schizophrenia (TRS) who have previously tried other antipsychotics at adequate doses (two or more, with at least one being atypical). However, despite optimal treatment, a subgroup of TRS patients with what is known as ultra-treatment-resistant schizophrenia (UTRS) fails to respond to clozapine, which occurs in 40–70% of cases. The most common approach to manage UTRS involves augmenting clozapine with pharmacological or non-pharmacological interventions, with a growing body of evidence that supports the use of electroconvulsive therapy (ECT) as an augmenter. This prospective non-randomized 8-week study, which followed the TRIPP Working Group guidelines and is one of few that separate TRS from UTRS, aimed to evaluate the effectiveness of clozapine in TRS patients and the efficacy of ECT augmentation of clozapine in UTRS patients. Patients with TRS were assigned to receive clozapine alone (clozapine group), whereas UTRS patients received bilateral ECT in addition to their current medication regimen (ECT plus clozapine group). The severity of symptoms was evaluated using the Clinical Global Impression Scale (CGI) and Positive and Negative Syndrome Scale (PANSS) at baseline and at the end of the 8-week trial. Both treatment approaches resulted in improved CGI and PANSS scores. The results suggest that both clozapine and ECT are effective treatment options for patients with TRS and UTRS, respectively, and that adherence to guidelines should provide a better frame for future clinical studies.

## 1. Introduction

Treatment-resistant schizophrenia (TRS) is a major cause of disability, affecting up to 34% of those diagnosed with schizophrenia [1]. The standard definition of TRS involves the failure of at least two non-clozapine antipsychotics. However, until recently, there was a lack of consensus regarding certain aspects of TRS. For instance, defining an adequate drug trial or therapeutic response was inconsistent in the literature [2,3,4]. Furthermore, distinguishing TRS from pseudo-resistance, which can stem from inadequate dosage or treatment duration, medication non-adherence, insufficient plasma levels of a medication, misdiagnosis, or adverse treatment events [5,6], presented further complications. To address these issues, the Treatment Response and Resistance in Psychosis (TRIPP) Working Group developed criteria for defining TRS [4]. These criteria stipulate that patients must not show significant improvement after treatment with at least two different non-clozapine antipsychotics for a minimum duration of 6 weeks and in a dose equivalent to a minimum of 600 mg of chlorpromazine per day. The TRIPP Working Group also provides guidance on adherence, advising that patients should adhere to their medication regimen at a rate of 80% or higher over a 12-week period; they propose using at least two of the following methods to assess adherence: pill counts, review of dispensing records, or reports from the caregivers. Finally, they recommend that functional impairment be included in the diagnostic criteria and measured using validated scales, such as the Positive and Negative Syndrome Scale (PANSS).

Despite some contrary opinions based on a network meta-analysis conducted by Samara et al. in 2016, clozapine is still considered the preferred treatment option for patients diagnosed with TRS [7,8,9]. Developed in 1958, it was the first atypical antipsychotic but was not extensively used due to reports of agranulocytosis in the 1970s, leading to its withdrawal from the market [10]. However, after a study by Kane et al. in the late 1980s showed its superiority in treating treatment-resistant schizophrenics, clozapine was later reintroduced [11]. Currently, it is the only FDA-approved antipsychotic for patients with TRS who have previously taken adequate doses of other antipsychotics, with a minimum requirement of a trial of two or more, with at least one being an atypical antipsychotic. However, even with optimal treatment, a significant proportion of patients with TRS, ranging from 40% to 70%, fail to respond to clozapine [12,13,14]. They form a distinct subgroup of TRS, referred to as clozapine-resistant schizophrenia (CRS). The TRRIP Working Group suggests that this condition should be considered a subspecifier of TRS and termed ultra-treatment-resistant schizophrenia (UTRS) due to the specific role of clozapine in treating TRS. In terms of defining an “adequate” clozapine trial, they propose a minimum of three months with serum levels above 350 ng/mL or a minimum dosage of 500 mg/day when obtaining blood samples is not feasible [4].

The management of UTRS remains a challenge in the field of psychiatry. The most common approach to address this condition is through augmentation with other medications, such as additional antipsychotics, anxiolytics, mood stabilizers, and antidepressants. However, despite these efforts, there is limited evidence to support the efficacy of augmentation with other medications [13,14,15,16,17,18,19]. Non-pharmacological interventions, including electroconvulsive therapy (ECT), repetitive transcranial magnetic stimulation (rTMS), and cognitive behavioral therapy (CBT), have emerged as alternative strategies to manage UTRS. ECT, in particular, has been used as a treatment modality since the 1930s [20]. It is a medical procedure that utilizes electrical stimulation to induce brief, controlled seizures in patients who are under anesthesia and have been given a muscle relaxant [21]. Numerous studies have investigated the effectiveness of ECT as an augmentation strategy [22,23,24]. For example, a prospective longitudinal observational study by Grover et al. found that ECT was an effective augmentation strategy for both CRS and non-CRS patients, as evaluated by PANSS ratings [23]. Lally et al. conducted one of the largest retrospective studies (*n* = 42) of the combination of clozapine and ECT in CRS, assigning CGI scores based on medical documentation, and reported a positive response rate of 76%, with 75% of responders not requiring hospitalization over the course of one year [24]. Another retrospective case series study by Kim et al. showed that ECT induced remission in 71.4% of patients with CRS [25]; although the sample size was small (*n* = 7) and the study lacked the use of CGI scores, it demonstrated promising results. In a high-quality study of individuals with clozapine-resistant symptoms, ECT augmentation resulted in 50% of participants achieving a response compared to 0% of participants who received clozapine without ECT augmentation; the sample included 39 participants (clozapine group, *n* = 19; ECT plus clozapine group, *n* = 20) [26]. A meta-analysis of 18 randomized controlled trials (*n* = 1769), conducted by Wang et al., concluded that ECT augmentation of clozapine in CRS is highly effective and relatively safe [27].

Overall, the available data implies that adjunctive non-pharmacological therapies such as ECT may prove to be beneficial in individuals with TRS/UTRS. Based on these data, we conducted a prospective non-randomized study to evaluate the effectiveness of clozapine in TRS patients and the efficacy of ECT augmentation of clozapine in UTRS patients. The goal was to see how two different groups of patients, previously differentiated by the TRIPP criteria, would respond to therapy. This study is one of the few that adheres to the TRIPP Working Group guidelines and distinguishes TRS from UTRS. Failure to differentiate between these two conditions can pose a significant challenge in defining the neurobiology of the disease. It seems reasonable to subtype based on treatment response, and studying the mechanisms leading to TRS and UTRS, as well as their differences, could offer an opportunity to develop novel treatments.

## 2. Materials and Methods

This was a non-randomized prospective study incorporating non-blinded treatment and blinded assessments. It included patients with treatment-resistant and ultra-treatment-resistant schizophrenia (TRS and UTRS) that were assigned to two treatment groups. Patients with TRS were introduced to clozapine (clozapine group) for 8 weeks, whereas the others received a course of bilateral ECT in addition to their current pharmacotherapy regimen (ECT plus clozapine group), also during an 8-week period.

### 2.1. Participants

All participants were recruited from the inpatient units of the University Hospital Center Sestre Milosrdnice in Zagreb, Croatia, between September 2020 and August 2022. The Ethics Committee of the University Hospital Center approved the study. All patients that fulfilled TRS and UTRS criteria were eligible for being part of this study. Accordingly, all the consecutive patients diagnosed with TRS and UTRS were approached upon hospitalization. After a complete and extensive description of the study profile, only subjects with signed informed consent forms were included. The forms were signed either by subjects themselves or by their legal tutor.

### 2.2. Inclusion Criteria

Patients of both sexes, aged between 18 and 65, were included. All of them fulfilled criteria for a DSM-5-TR diagnosis of schizophrenia based on a clinical interview and follow-up of experienced psychiatrists from the Sestre Milosrdnice University Hospital Center. Additionally, they also fulfilled TRRIP criteria for either TRS or UTRS. All subjects or a legal tutors signed an informed consent form.

Treatment-resistant schizophrenia (TRS) was defined according to TRIPP criteria:

(1) Determination of treatment non-response. Defined as <20% symptom reduction over ≥6 weeks. For evaluation, we used the Clinical Global Impression Scale (CGI) and the Positive and Negative Syndrome Scale (PANSS) [28,29].

(2) Determination of treatment resistance. Defined as non-response to ≥2 adequate treatment trials. In our study, each subject has had a history of at least two failed trials of ≥600 mg of chlorpromazine equivalents for at least 6 weeks.

(3) Determination of adequate treatment trial. Defined as at least a 6-week trial at a therapeutic dose equivalent to ≥600 mg chlorpromazine daily.

(4) Determination of adherence. Defined as ≥80% prescribed doses taken. In our study, this was assessed with the following methods: pill counts and dispensing chart reviews. Obtaining an antipsychotic blood level at least once is also requested, which was performed without advanced warning.

Ultra-treatment-resistant schizophrenia (UTRS) meets all of the criteria above, with the addition of non-response to adequate trial on clozapine. After plasma levels reach 350 ng/mL, a trial should last at least 3 months. Our subjects with UTRS fulfilled all of the suggested criteria.

### 2.3. Exclusion Criteria

Individuals with a history of epilepsy, ECT treatment within 6 months prior to enrollment in the study, severe neurological or systemic disorders that could significantly impact cognition, behavior or mental status (excluding tardive dyskinesia or neuroleptic-induced parkinsonism) within 3 months prior to the study, and psychoactive substance abuse (excluding nicotine or caffeine) within 1 month prior to the study were excluded from participation.

### 2.4. Medications

Participants in both groups were on clozapine either in monotherapy or in combination with other psychotropic drugs. Concurrent use of other antipsychotic, antidepressants, or anticonvulsants was allowed as long as they were taken at a stable dose for at least 12 weeks before entering the study. Both groups were similar in terms of the types of drugs used, the main difference between them was that patients in the clozapine group were newly initiated on clozapine, whereas those in the ECT plus clozapine group had previously received clozapine.

In the clozapine group, patients were allowed, based on the decision of their treating physician, to remain on their previous psychopharmacological medications, primarily antipsychotics, if they had a different mechanism of action than clozapine. Seven patients were using risperidone, five were using aripiprazole, three were using haloperidol, and one was using cariprazine in this group. Four patients in this group were also using valproate, but none of them were taking a combination of two antipsychotics and valproate.

In the ECT plus clozapine group, all patients had previously been treated with clozapine and continued to use it during the study. Of the 36 participants, 6 were using a combination of clozapine and risperidone, 5 were using a combination of clozapine and haloperidol, 5 were using a combination of clozapine and aripiprazole, and 3 were using a combination of clozapine and cariprazine. In terms of other medications, three patients were taking low doses of escitalopram.

### 2.5. ECT Procedure

The Thymatron System IV (Somatics, Lake Bluff, IL, USA) was used for bilateral ECT with a standard brief pulse stimulus threshold titration and dosing [30]. To induce anesthesia, propofol (1 to 2 mg/kg) was used, and muscle relaxation was achieved using succinylcholine (0.5 mg/kg) with atropine 0.5 mg intravenously. The ECT treatment protocol involved three sessions per week for the initial four weeks, followed by twice-weekly sessions for the next four weeks. If patients met remission criteria before the completion of eight weeks and showed a plateau in their improvement for two consecutive ratings, they continued to receive ECT weekly until the end of eight weeks. Before the start of the ECT treatment, all subjects or their legal guardians provided written consent for the procedure.

### 2.6. Assesments

Eligible, consenting patients had a complete baseline medical and psychiatric evaluation. Patients were rated at baseline and after trial completion (end of week 8 for both groups). The severity of symptoms was evaluated by the CGI and PANSS [28,29].

### 2.7. Blindness

Raters were blinded for a patient’s group status and received treatment.

### 2.8. Statistical Analysis

To assess the normal distribution of the data, we utilized the Kolmogorov–Smirnov test. We presented the demographic and clinical characteristics of the two groups as percentages, as well as mean ± standard deviation (SD). We employed a repeated measures analysis of variance (RM ANOVA) to examine the PANSS scores at baseline and at week 8, while also adjusting for confounding covariates. Between-group factors (clozapine and ECT augmentation) and within-group factors were set. Additionally, we conducted an RM ANOVA to measure CGI scores. To rule out type I errors, we employed the Bonferroni correction as we had five different variables in the same model (Bonferroni corrected *p*-value = 0.05/5 = 0.01). We considered a difference to be statistically significant if the *p*-value was less than 0.0002. The statistical analysis was carried out using SPSS software (version 20.0, SPSS, Chicago, IL, USA).

## 3. Results

### 3.1. Baseline Data

Out of the 103 patients who met the inclusion criteria, 74 individuals (71.8%) provided their consent to participate in the study. The clozapine group consisted of 35 patients, whereas 39 patients met the inclusion criteria for the UTRS (clozapine plus ECT group). In the clozapine group, 33 out of the 35 patients completed the 8-week clozapine phase, with two patients dropping out due to their refusal to continue participating in the study while there was no change in their treatment. In the clozapine plus ECT group, 36 out of 39 participants completed the 8-week trial, whereas three individuals dropped out early, refusing further ECT treatments.

The mean age of patients in the clozapine group was 55 ± 9 years, whereas the mean numbers of episodes and hospitalizations were 4.5 ± 2 and 5 ± 5, respectively. On the other hand, the mean age of patients in the ECT augmentation group was 38.5 ± 13 years, whereas the mean numbers of episodes and hospitalizations were 8 ± 5, and 6 ± 3, respectively. The mean duration of illness was 211 ± 136 months for the clozapine group and 128 ± 108 months for the clozapine plus ECT group. Other demographic characteristics are shown in Table 1. Overall clinical characteristics (baseline and week 8 CGI, baseline and week 8 PANSS) are shown in Table 2. The average doses of clozapine in the clozapine group and the clozapine plus ECT group were 223.5 mg/day and 571.4 mg/day, respectively. The corresponding mean plasma levels of clozapine were 477.5 ng/mL and 659.9 ng/mL.

### 3.2. Clinical Response

#### 3.2.1. CGI Scores

Statistical analysis showed a significant difference between the groups in initial Clinical Global Impression (CGI) scores (Wilks lambda = 0.112, F(1.68) = 520.858, *p* < 0.001, partial eta squared = 0.888). However, there was no significant interaction between the type of intervention and time, indicating that both groups showed similar improvements in CGI scores over time (Wilks lambda = 0.990, F(1.68) = 0.664, *p* = 0.418, partial eta squared = 0.010). After Bonferroni correction, the separate influence of the two types of interventions was not significant (F(1.68) = 0.664, *p* = 0.418, partial eta squared = 0.010), suggesting that both clozapine and ECT augmentation of clozapine are equally effective in improving CGI scores in TRS and UTRS patients.

#### 3.2.2. PANSS Positive Symptom Subscores

After comparing the initial values, it was determined that there was a statistically significant difference between the groups (Wilks lambda = 0.331, F(1,68) = 135.495, *p* < 0.001, partial eta square = 0.669). A significant interaction between time and type of intervention was observed (Wilks lambda = 0.787, F(1.68) = 18.157, *p* < 0.001, partial eta squared = 0.213), with both groups showing an improvement in positive symptom subscores. The separate influence of the two types of intervention was also significant (F(1.68) = 18.157, *p* < 0.001, partial eta square = 0.213), indicating that clozapine and ECT augmentation of clozapine are NOT equally effective.

#### 3.2.3. PANSS Negative Symptom Subscores

By comparing the initial values, a statistically significant difference between the groups was determined (Wilks lambda = 0.266, F(1.68) = 184.690, *p* < 0.001, partial eta square = 0.734). There was no significant interaction between the type of intervention and time (Wilks lambda = 0.895, F(1.68) = 7.901, *p* = 0.006, partial eta squared = 0.105), where both groups recorded an improvement in negative symptom subscores; the separate influence of the two types of intervention was significant, F(1.68) = 7.901, *p* < 0.006, partial eta square = 0.105, which means that both interventions are NOT equally effective.

#### 3.2.4. PANSS General Psychopathology Subscores

A comparison of the initial values revealed a statistically significant difference between the groups (Wilks lambda = 0.275, F(1.68) = 176.795, *p* < 0.001, partial eta square = 0.725). There was no significant interaction between the type of intervention and time (Wilks lambda = 0.852, F(1.68) = 11.659, *p* = 0.001, partial eta squared = 0.148), where both groups recorded an improvement in general psychopathology subscores; the separate influence of the two types of intervention was significant, F(1.68) = 11.659, *p* = 0.001, partial eta square = 0.148, which means that both interventions are equally effective.

#### 3.2.5. PANSS Total Scores

By comparing the initial values, a statistically significant difference between the groups was determined (Wilks lambda = 0.202, F(1.68) = 264.891, *p* < 0.001, partial eta square = 0.798). A statistically significant influence of time was determined (Wilks lambda = 0.779, F(1.68) = 18.987, *p* < 0.001, partial eta squared = 0.221), where both groups recorded an improvement measured in total scores; the separate influence of the two types of intervention was significant, F(1.68) = 18.987, *p* < 0.001, partial eta square = 0.221, which means that both interventions are NOT equally effective.

### 3.3. Side Effects

No significant differences in side effects were observed between the two groups in relation to any of the rated symptoms. Additionally, there were no ECT-related side effects observed during the study. This was expected as the stimulus intensity required to exceed the seizure threshold was lower (40% max), and therefore, any potential adverse effects were minimized.

## 4. Discussion

Clozapine has been widely recognized as the gold standard treatment for TRS due to its superior efficacy in improving positive, negative, and cognitive symptoms, as well as reducing the risk of suicidal behavior and hospitalizations. In addition to clozapine, ECT has been increasingly recognized as an effective augmentation strategy and remains the oldest biological treatment used in modern psychiatry. The present study’s findings are consistent with previous case reports and studies that have suggested the effectiveness of both clozapine and ECT in the management of TRS and UTRS patients [12,26,31,32,33,34,35,36]. For instance, McEvoy et al. (2006) found that clozapine was more effective than olanzapine in treating TRS [31]. Lewis et al. (2006) and Masoudzadeh et al. (2007) reported that ECT was effective in augmenting clozapine in UTRS patients [32,33]. One review found that clozapine was more effective in reducing symptoms of schizophrenia in treatment-resistant patients compared with other antipsychotic medications; the author also noted that the use of clozapine was more cost-effective than other treatments [34]. In another systematic review that evaluated the use of ECT as an augmentation therapy for antipsychotic medication in patients with TRS, ECT was found to be effective in reducing the symptoms and improving overall functioning in patients [35]. Only one network meta-analysis has challenged the superiority of clozapine [7]. The authors of that study noted the need for clear guidelines regarding therapeutic resistance and patient adherence. Our study is one of the few that follows the TRIPP Working Group guidelines and differentiates between TRS and UTRS. Our primary objective was to explore the therapeutic responses of these two distinct groups of schizophrenia patients, previously differentiated by the TRIPP criteria. In conclusion, our study’s results contribute to the growing evidence supporting clozapine and ECT’s effectiveness in treating TRS and UTRS patients, respectively.

The definitions of TRS and UTRS are outlined in guidelines but are underrepresented in clinical trials. This distinction is crucial for understanding the neurobiology of the disease, as these two forms of illness may have distinct pathological and pathophysiological characteristics. Studies that have directly compared TRS with UTRS or UTRS with healthy control individuals have shown that UTRS is associated with lower prefrontal perfusion, smaller thalamic volume, higher anterior cingulate gyrus glutamate levels, low-grade peripheral inflammation, and weaker network connectivity [37,38,39,40,41,42]. Therefore, subtyping based on treatment response is a viable approach, and understanding the mechanisms leading to TRS and UTRS, as well as the difference between them, may pave the way for the development of novel treatments.

To enhance our comprehension of TRS and UTRS, it is essential that independent studies adhere to standardized and objective criteria, listed in various guidelines. UTRS is generally defined as a lack of appropriate response to the drug with clozapine plasma levels of 350 ng/mL or above and a minimum duration of clozapine therapy of 8–12 weeks after achieving therapeutic plasma levels, according to several schizophrenia guidelines such as the TRIPP Working Group, the World Federation of Societies of Biological Psychiatry (WFSBP), and the Canadian guidelines for the Pharmacotherapy of Schizophrenia in Adults. The recommended clozapine dosage varies among guidelines, ranging from 100 to 900 mg/day. Given that the lack of consistent or absent definitions in publications has impeded the acquisition of high-quality evidence for the treatment of UTRS, adhering to guidelines is crucial for future clinical studies to have a better framework.

Evaluating the baseline parameters of our subjects, we encountered a moderately ill population in the ECT augmentation group and a markedly ill one in the clozapine group (see Table 2). However, although the ECT augmentation group had lower PANNS scores, it was much younger and had a greater number of episodes/hospitalizations, suggesting a more severe form of the disease. In a prospective study of a Brazilian population, by comparing TRS and non-TRS patients the authors determined that a lower baseline PANSS score was predictive of TRS [43]; in our study, compared with TRS, the same can be said about UTRS patients. We also found that UTRS patients have a more urban residence, which is in correlation with past evidence of urban areas increasing the risk of schizophrenia [44]. Furthermore, compared with TRS, our UTRS patients are more often unemployed and use drugs more frequently. In this study, there were no differences in side effects between patients receiving ECT augmentation therapy and the clozapine group.

Our study was designed with several key considerations in mind. Firstly, we focused on patient selection. By using consensus guidelines and the recommendations of the TRRIP Working Group, we ensured that our study was comparable with others and could be replicated in future studies. So far, the lack of consensus in defining or diagnosing TRS was likely an important reason why many findings have not been replicated and have conflicted with each other. Another important aspect of our study was the size of the sample. Over a period of nearly two years, we were able to obtain an acceptable sample size, with two evenly matched groups in terms of the number of participants. Additionally, we used widely accepted rating scales, such as the CGI and PANSS, to provide appropriate efficacy assessments.

However, our study also has several limitations. Firstly, it was non-randomized and included only Caucasian inpatients. Future studies could benefit from using a randomized design and a sample that includes participants from other ethnicities. Secondly, we did not conduct statistical analysis of the demographic differences between groups. Instead, we treated them as two distinct cohorts with two distinct forms of illness. Thirdly, almost all of our patients were prescribed additional psychotropic medications in addition to clozapine. This may have confounded our results, making it difficult to determine the specific effects of clozapine and ECT augmentation therapy. Lastly, our study only followed patients for a short period of time, which prevented us from drawing conclusions about the long-term effects of clozapine and ECT augmentation therapy in this sample.

## Figures and Tables

**Table 1:** Demographic and Clinical Characteristics of Patients by Treatment Group.

	Clozapine + ECT		Clozapine	
	*n*	%	*n*	%
Sex				
male	23	64	12	36
female	13	36	21	64
Education				
completed prim. education	5	13.9	4	12.1
completed sec. education	26	72.2	20	60.6
completed higher education	5	13.9	9	27.3
Area				
urban	34	94.4	20	60.6
rural	2	5.6	13	39.4
Marital status				
never married	24	66.7	17	51.5
married	9	25	6	18.2
divorced	2	5.6	9	27.3
widowed	1	2.8	0	0
extramarital union	0	0	1	3
Labor status				
employed	5	13.9	6	18.2
unemployed	25	69.4	9	27.3
retired	6	16.7	18	54.5
Suicidality				
yes	10	27.8	10	30.3
no	26	72.2	23	69.7
Suicide attempts				
yes	3	8.3	5	15.2
no	33	91.7	28	84.8
Psychiatric disorders in family history				
yes	15	41.7	12	36.4
no	21	58.3	21	63.6
Drugs				
yes	6	16.7	1	3
no	30	83.3	32	97
Alcohol				
yes	3	8.3	6	18.2
no	33	91.7	27	81.8
Tobacco				
yes	16	44.4	16	48.5
no	20	55.6	17	51.5

**Table 2:** Clinical Outcomes of Patients by Treatment group.

	Clozapine + ECT		Clozapine		*p*-Value
	M	SD	M	SD	
CGI					
CGI baseline	5.7	0.8	5.4	0.8	<0.001
CGI week 8	3.2	0.9	3.2	0.8	0.418
PANNS					
P subscore baseline	17	7	23	6	<0.001
P subscore week 8	12	4	13	4	<0.001
N subscore baseline	25	7	27	8	<0.001
N subscore week 8	20	6	19	6	<0.006
G subscore baseline	46	11	50	8	<0.001
G subscore week 8	37	9	34	8	0.001
total score baseline	87	20	100	17	<0.001
total score week 8	68	17	67	15	<0.001

CGI Clinical Global Impression; PANNS Positive and Negative Syndrome Scale.

## Data Availability

The data presented in this study are available on request from the corresponding author. Due to privacy and ethical restrictions, the data underlying this study cannot be made publicly available.
